# Next Steps: Applying a Trauma-Informed Model to Create an Anti-Racist Organizational Culture

**DOI:** 10.3390/bs12020041

**Published:** 2022-02-09

**Authors:** Nina Esaki, Maxine Reddy, Cameron T. Bishop

**Affiliations:** 1 Springfield College, 263 Alden St, Springfield, MA 01109, USA; 2 Andrus Sanctuary, 1156 North Broadway, Yonkers, NY 10701, USA; mreddy@jdam.org; 3 The Methodist Home, 304 Pierce Ave, Macon, GA 31204, USA; Cameron.Bishop@themethodisthome.org

**Keywords:** trauma-informed care, trauma-responsive care, cultural humility, anti-racism, DEI, organizational change, Sanctuary Model

## Abstract

Although there has been a significant increase in the delivery of evidence-supported, trauma-informed care over the past few years, there has been less discussion around the consideration of the broader cultural, political, and societal factors that contextualize client trauma and that also need to be recognized and understood to promote healing and prevent future trauma. In support of sharing some best practices and lessons learned, this article provides a case study of one agency that has used the Sanctuary Model^®^, an evidence-supported, trauma-informed organizational change model, to introduce the practice of cultural humility with staff as a facilitator of improved service delivery for clients from culturally marginalized communities. The model supports these endeavors through the adherence to the seven commitments, a set of organizational values for creating a trauma-informed community, allowing for all voices to be heard and considered and providing opportunities to begin the repair of previous experiences of inequity and suppression. Through the board of directors, leadership, and staff, the organization transformed its culture into one that truly supports and embraces diversity, equity, and inclusion in its operation for the benefit of both staff and clients alike.

## 1. Introduction

The murder of George Floyd in Minneapolis, Minnesota in May 2020 spurred a reawakening in the United States to racial trauma and long-standing historical challenges around racial justice and equality. Ever since the founding fathers laid the foundation for the birth of our nation on the backs of slaves, by tacitly acknowledging the institution of slavery in the U.S. Constitution, there has been historical trauma for Blacks in this country [1]. Despite the Thirteenth Amendment to our Constitution, ratified in December 1865 to formally abolish slavery, there have been a number of federal, state, and local laws enacted that have continued to hamper the equitable treatment of Blacks in our communities. 

When the Social Security Act and other New Deal welfare programs were enacted in the 1930s, they excluded Black farm laborers and domestic workers—the great majority of Black workers at that time [2]. More recently, the Voting Rights Act of 1965 that ushered in a new era of democratic participation [3] is being curtailed by state lawmakers in several states to limit access to voting by communities of color [4]. These long-standing structural impediments to equitable treatment under the law cause profound and intergenerational harm to many of our citizens.

Reinforced in light of COVID-19 and civil unrest, there is a renewed focus on intentionally naming anti-racism, the process of actively identifying and opposing racism [5], and anti-oppression within the overarching umbrella of a trauma-informed approach. Without looking beyond the lens of individual trauma, professionals, organizations, and systems risk retraumatization of individuals and communities who have experienced trauma resulting from interpersonal, systemic, and structural racism and oppression [6,7].

## 2. Historical Trauma

Historical trauma is characterized as a traumatic event shared by a group of people that contributes to an increased prevalence of negative physical health outcomes, distrust, and mental illness in subsequent generations [8]. It has been experienced by many, including the First Nations people, slaves and their descendants, Jewish Holocaust survivors and descendants, Japanese-American internment camp survivors and descendants, and other exploited and persecuted populations [9]. 

The First Nations people experienced repeated massacres and the forced removal of children to federal and mission boarding and day schools [10]. A history of human trafficking, systematic dehumanization, racism, discrimination, prejudice, and stereotyping has contributed to a cumulative and collective sense of pain and distress for many persons of African ancestry in the United States on a conscious and/or subconscious level [11,12,13]. From September 1939 to May 1945, the population of European Jews was reduced from about 8 million to approximately 1.6 million. Although the traumatic events of the Nazi Holocaust happened more than half a century ago, they still affect the lives of survivors and subsequent generations [14]. Also, the World War II internment of more than 120,000 Japanese-Americans represents one of this country’s most striking examples of social injustice [15]. For members of any of these communities, daily reminders of racial discrimination can exacerbate individual responses to trauma [16]. 

Racism is not only about individual acts of meanness or prejudice, but refers to a systemic power arrangement that permeates most institutions in American society [17]. Some individuals advocating for a multiracial, anti-racist society view racism as a structural/political reality that functions to maintain all other forms of oppression. From their perspective, movements to eliminate “isms” such as classism, sexism, homophobia, ageism, and ableism cannot succeed unless racism is first addressed. For as long as racism exists, the elimination of other “isms” would only benefit white people; yet those of color who are poor, or who are women, or gay or lesbian, etc., will continue to face their particular forms of oppression in addition to facing racism [17]. The problem of racism is not housed in any single entity, but in the complex interlocking of policies and institutions that reinforce one another. Pinderhughes [18] states, “Belief in superiority of Whites and the inferiority of people-of-color based on racial differences is legitimized by societal arrangements that exclude the latter from resources and power and then blame them for their failures, which are due to lack of access” (p. 89).

Racial trauma, or race-based stress, refers to the events of danger related to real or perceived experiences of racial discrimination [19]. There include threats of harm and injury, humiliating and shaming events, and witnessing harm to other people of color that was due to real or perceived racism [20]. There has been a recent rise in hate crimes [21], with Blacks being more exposed to racial discrimination than other ethno-racial groups [22].

## 3. Trauma-Informed Care

Cultural awareness, responsiveness, and understanding are essential to increasing access and improving the standard of trauma-informed care for children, families, and communities. Trauma-informed systems acknowledge the compounding impact of structural inequity and are responsive to the unique needs of diverse communities as represented by culture, history, race, gender, location, and language. Given the systemic roots of inequities, truly trauma-informed services require culturally responsive involvement across organizations, communities, and service sectors to reduce barriers, overcome stigma, address social adversities, and promote positive ethnic identities [23].

The examination and integration of historical and social contextual factors are necessities when providing community psychological health services to persons of African ancestry in the United States [24,25]. The existence of ongoing stress, such as institutional racism, can exacerbate a person’s post-traumatic reactions. Therefore, it is essential to work with a lens based in trauma theory that acknowledges forms of oppression, such as racism and poverty. The lens must also include methods by which oppression in not perpetrated. Effective intervention must acknowledge the impact of trauma and recognize racism and poverty as potential sources of traumatic injury. Furthermore, residential treatment must not itself be a source of trauma, including the trauma of racism and oppression [26]. 

The context in which service providers work, including how they are treated by leaders and colleagues in the organization, has an impact on how they are able to deliver high quality services. African-Americans’ employment functioning is also affected by their experiences of racism [27]. Alleyne [28,29] conducted two studies of African-Americans’ experiences with racism in the workplace and participants shared that they often deferred to White European-American colleagues, and tended to wait for opportunities to show their abilities, rather than asserting themselves. Alleyne’s [28,29] research supports Wilson’s [30] theory that African-Americans unconsciously rely on the White oppressor to undo their oppression, and if left unchecked, this may exacerbate continued traumatic oppression.

To mitigate the existence of ongoing stress, such as institutional racism, in our residential care facilities, it is essential to work from a trauma theory perspective, which acknowledges forms of oppression, such as racism [26]. Organizations can experience trauma, just like individuals [31]. The trauma can be from external or internal events, even from deleterious effects of dysfunctional internal dynamics that develop over time. In response to these forces, organizations may become redemptive organizations [32] and/or develop reparative cultures [33]. To work toward an anti-racist system, it is necessary to define the areas in which racism and White privilege are present, and all in the facility, staff and clients alike, must have an opportunity to develop healthy and well-integrated racial identities. 

## 4. Cultural Humility

An often overlooked but fundamental principle of a trauma-informed approach involves cultural humility [34]. Cultural humility through culturally competent practices (e.g., acknowledgement of diverse values, beliefs, and behaviors) supports the understanding of the multilayered intersection between trauma and aspects of culture including race, ethnicity, gender, geographic location, socio-political particularities, and language [35,36]. Unfortunately, current guidelines in trauma-informed approaches do not sufficiently account for cultural humility as a facilitator of service delivery and engagement in working with ethnic/racial minorities. As evidenced by the findings of a systematic review by Hanson and Lang [37] on the principal components of trauma-informed approaches from well-established frameworks, such as SAMHSA, cultural humility did not emerge as a core component, nor did the significant role of structural inequities on traumatic exposure or service access [35]. 

In Gottlieb’s [38] recent literature review on cultural humility, she presents a distilled framework including three principles, namely: “(1) committing oneself to an ongoing process of compassionate self-awareness and inquiry, supported by a community of trusted and cognitively-diverse colleagues; (2) being open and teachable, striving to see cultures as our clients see them, rather than as we have come to know or define them; and (3) continually considering the social systems—and their attendant assignations of power and privilege—that have helped shape reality as both we and our clients experience it” (p. 3). 

On an institutional level, cultural humility asks that we interrogate identities that are culturally dominant or that have been assigned privilege with an equal curiosity as toward those that have been marginalized, to examine all identities with a critical lens, and to be vigilant to ways in which our workplaces reinforce or dismantle existing power structures [38]. For an organization to be truly trauma-informed, it is essential that it be committed to a culture of diversity, equity, and inclusion [6].

## 5. Sanctuary Model

One evidence-supported, trauma-informed organizational change intervention that has offered agencies a framework to advance an anti-racist organizational culture is the Sanctuary Model^®^ [26,39]. The Sanctuary Model is an organizational culture intervention designed to support and facilitate the development of structures, processes, and behaviors that can counteract traumatic experiences or extended exposure to adversity [40]. The Sanctuary Model provides organizations with a blueprint for creating trauma-informed communities through organizational change efforts [41,42]. Created by Bloom, along with her colleagues Foderaro, a clinical social worker, and Ryan, a clinical nurse practitioner, the Sanctuary Model is an organizational change model born from their work in a psychiatric inpatient hospital for adults [43,44]. The Sanctuary Institute at Andrus is the training and consulting home of the Sanctuary Model, having developed specific training and implementation milestones, and it is currently focused on delivery to and support for human services organizations.

This model defines sanctuary as a place of temporary refuge that allows for a different kind of social experience, where some of the usual societal rules are suspended and where the culture promotes safety not only for the clients, but for the staff as well [45]. The Sanctuary Model is a full systems approach to changing organizational culture [41]. Bloom [46], through this model, thinks that the primary component that leads to change is the creation of a safe, nonviolent community that promotes recovery for all individuals, and helps survivors of trauma and chronic stress to move past the effects of the trauma and stress by rebuilding and creating healthy attachments. Community in this respect refers to an organization and to departments within an organization, usually in the business of human services. This approach utilizes specific structures, practices, and behaviors to transform an organization [42]. 

Using four pillars as a foundation, the Sanctuary Model offers a lens for understanding behavior, both individual and organizational, as a manifestation of chronic and overwhelming experiences. 

The four pillars are trauma theory, the seven commitments, the SELF framework, and the Sanctuary tools. **Trauma theory** provides a focus on how traumatic events affect the mind through repression and subsequent re-enactment [47]. Re-enactment is the act of repeating patterns of past behavior from unresolved traumatic experiences. Trauma theory also informs that there are biological, psychological, and social effects of traumatic events, or events that are perceived as traumatic. Trauma theory, as a pillar of the Sanctuary Model, exposes staff to an overview of the effects that traumatic experiences have on individuals, organizations, and systems. 

The **seven commitments** focus on creating a safe and healthy environment by offering a set of organizational values that promote healing from trauma and adversity, thereby shaping a culture that directly mitigates their impacts. The seven commitments of Sanctuary are designed as a way to lead individuals and organizations away from trauma-reactive behaviors [42] and mitigate the effects of trauma. The seven commitments provide reparative experiences for all and create a safe community, not only in interactions with each other, but in approaching the work of the organization and planning and assessing individual and organizational progress. 

The seven commitments are:

- Nonviolence—creating a culture of safety, physically, socially, psychologically, and morally;
- Emotional intelligence—increasing skills in identifying one’s own emotions and the emotions of others, understanding the relationship between past experiences and current emotions and behaviors;
- Social Learning—the promotion of collaborative thinking and problem solving, with a belief that everyone has something to offer. Mistakes are viewed as learning opportunities;
- Democracy—to mitigate the effects of helplessness and prevent learned helplessness in the future, this Commitment requires everyone to have a voice and for leaders to make the best decisions based on everyone’s input;
- Open communication—the antidote to secrecy that is often experienced by those who have endured traumatic events, creating a community that tolerates the expression of emotions;
- Social responsibility—a commitment to social responsibility is a focus on building a community in which people feel a sense of care for each other and as a group. Social responsibility holds people accountable for their actions; and
- Growth and change—a focus on hope and on the future. This commitment encourages people and organizations to move away from potentially getting stuck in the past, thus preventing healing and growth, and focusing on setting achievable goals on a regular basis.

The **SELF framework** is a powerful structure that helps to support innovation and guide conversations through the simple and accessible language of safety, emotion management, loss, and future, and is used to solve system and organizational problems in a fluid fashion that can also appreciate the complex issues faced by organizations. These four categories represent the areas of dysfunction in individuals and in systems exposed to trauma and adversity, as well as the four areas for targeted intervention and measurement of recovery. This framework provides a trauma-informed way of organizing conversations and documentation in a simple and accessible language. The SELF framework levels the playing field for clients, families, staff, and administrators by moving away from jargon and toward a more fundamental organizing system. These four categories represent the four dynamic areas of focus for trauma recovery. The SELF framework is also used to solve system and organizational problems in a nonlinear fashion that appreciates complexity. 

Safety is viewed from four areas. Physical safety includes intolerance of violence of any type, an absence of self-destructive behavior, and an avoidance of risk-taking behavior. Psychological safety focuses on self-protection, self-efficacy, and self-discipline, ensuring that people are making safe and positive decisions for themselves. Social safety refers to safety with others and focuses on safe attachments with individuals and in groups and the exercise of responsible authority. Moral safety focuses on being able to make decisions in line with a moral compass and being able to own up to one’s mistakes.

Emotion management encourages the trading of actions for words, building one’s capacity to contain and manage one’s own emotions rather than acting them out in potentially destructive ways. Emotion management is about recognizing the feelings of not only oneself, but of others, and recognizing the impact of one’s actions on others.

Loss refers to taking the time to mourn and acknowledge that loss impacts individuals and organizations. The Sanctuary Model acknowledges that any change creates a loss and needs to be reconciled. Focusing on loss also helps us to disrupt dysfunctional patterns and plan a new future with possible gains.

Focusing on future creates hope and support for changing trajectories, guiding toward new attractors, and making different choices. Imagination, creativity, and innovation help to create a different future for individuals and organizations.

Last, the Sanctuary Model offers a **tool kit** of trauma-informed practices, practical and simple interventions that reinforce the language and philosophy of the model. These tools promote skill development, enabling organizations and people to create shared values, manage difficult situations, and build common standards of practice in how their business is conducted.

The Core Team, as referenced by the organization below, is the vehicle that directs the implementation of the Sanctuary Model throughout the organization. The Core Team is a diverse mix of staff members representing all facets of the organization, including position, demographics, and the ability to influence others. In a high-functioning Sanctuary organization, the Core Team not only moves the implementation of Sanctuary forward, but reviews all initiatives undertaken by the organization with a trauma-informed lens, providing feedback and recommendations to leaders and boards of directors.

The Sanctuary Model has been used in the past to support the building of organizational cultures that promote anti-racism [26,39]. The organization selected for this manuscript has implemented the Sanctuary Model and has attained Sanctuary certification through the Sanctuary Institute. Implementation of the model is typically a three-year process supported by a faculty consultant from the Sanctuary Institute. Over the three years, the organization, as directed by the Core Team, will undertake numerous tasks that ultimately shift the organizational culture through a change in beliefs and behaviors, and will provide opportunities for introspection and discourse around policies and procedures that affect organizational functioning, the staff, and those who receive services. Sanctuary certification designates an organization as adhering to the Standards of Certification [48]. These standards are based on the four pillars of the Sanctuary Model and require both documented and experiential achievement of each of the 28 standards. Certification in the Sanctuary Model indicates that an organization has a strong trauma-informed culture that produces consistently high outcomes, and is validation of the efforts that have gone into creating a trauma-responsive culture. The Methodist Home for Children is one of these organizations and has begun an organizational journey to address and eliminate racism using the Sanctuary Model as the foundation.

## 6. The Methodist Home

Founded in 1872 and located in the Southeastern United States, The Methodist Home has a long history of operating through significant political and social changes, especially with regards to race. For many decades there had been Black staff members, but they were only assigned roles in direct services. It was not until the early 1980s that the organization hired a Black male for a leadership position. The years that followed continued to create opportunities for growth and change in the organization, much of it focused on creating a culture whereby all staff members are treated equally.

The Methodist Home received Sanctuary certification in June 2017. The organization was initially drawn to the model because of its emphasis on creating organizational cultures that are trauma-informed. Achieving certification required the agency to develop and practice new ways of thinking, interacting, and operating. Having found success in managing a variety of organizational challenges, confidence was high that utilizing the Sanctuary principles was an effective model. However, it was not until the murder of George Floyd that the model was truly tested. 

Peaceful protests, violent protests, and riots filled media outlets and social media feeds. Organizations small and large across the country entered the conversation, many issuing statements against racism or in support of Black Lives Matter. Employees of The Methodist Home were talking about the events as they unfolded and began asking the question, “What are we going to do?”

Rather than rushing into issuing a statement or making changes, the leadership team of the organization decided to trust in the pillars of the Sanctuary Model as a guide forward and asked the Core Team to assume the initial responsibility of developing a plan. The Core Team, a fundamental component of the Sanctuary Model consisting of approximately 30 individuals from across the organization, assembled with the purpose of beginning a conversation around how the public events were impacting people within the organization. Utilizing the SELF framework, the team began sharing both how the current events were impacting them and their own personal experiences of discrimination within and outside of the organization. Several common themes began to emerge that included “we must respond, this has gone on too long” and “do not just tell me what you are going to do, show me through your actions”. It became clear that the outcry following Floyd’s murder was not just an organizational issue; it was personal and rooted in both personal and systemic trauma.

The Core Team authorized the creation of a focused workgroup to begin developing a path forward. Naturally, several members of the Core Team volunteered to join the workgroup; however, other key centers of influence regardless of position on the organizational chart were asked to participate. Additionally, the group makeup was intentionally diverse in race, gender, and job role. The group became known as “Next Steps”, simply because the subject for the initial emails coordinating the group were titled, “Next Steps.” Although the group was intentionally selected by the Core Team, the members acknowledged that prior to making any recommendations for organizational change, they needed to create opportunities for organizational learning whereby any interested staff member could have a voice.

Drawing upon the principles of the seven commitments and the SELF framework, several mechanisms were implemented to intentionally provide staff a safe environment wherein they could share their emotions and experiences, especially as they related to racism, discrimination, and privilege. Acknowledging that there may be safety in remaining anonymous, a staff survey was developed and electronically distributed to the entire organization. The organization committed to analyzing the results and sharing the findings with all staff, regardless of the outcome.

The response rate to the staff survey surpassed all previous agency-wide surveys. The Next Steps workgroup reviewed the data, looking for common themes and questions. Several key areas emerged: hiring and promotion opportunities, pay, and “in-group” vs. “out-group” dynamics. Additionally, questions about organizational structure and board composition emerged. Open-ended questions provided feedback ranging from staff members not experiencing any forms of discrimination to staff members recounting specific incidents whereby they felt mistreated. 

To provide an opportunity for more personal interaction and learning, the Next Steps workgroup hosted two “listening sessions” for all staff. The purpose of these sessions was to provide an open forum wherein staff members could share their thoughts, experiences, or concerns with members of the leadership team. To increase the levels of transparency and open communication, the board chair was also invited and attended the sessions. With the hope of minimizing power dynamics, two external members of the community were asked to moderate the discussion. To help the moderators prepare for the sessions, they were provided with the raw data from the staff survey. Upon reviewing the data, the moderators advised that to maximize time and avoid getting side-tracked, the focus of the conversation should center on systemic forms of racism, discrimination, and white privilege rather than on conversations speaking specifically to issues such as pay scales. 

The moderated sessions were well-attended and the feedback provided indicated the participants felt safe in expressing personal and traumatic experiences both within and outside of the organization. In fact, several Black staff members stated that being able to share their experiences in this context was cathartic. Likewise, several White staff members stated that hearing the accounts of their Black colleagues began to create a new lens through which they could view diversity, equity, and privilege. Multiple staff members shared their personal experiences and observations of how the agency has managed a multitude of issues surrounding equity. Several individuals described experiences within the organization that had taken place decades prior, yet continued to impact their work today. A staff member was designated as a “scribe” for the sessions and used a large whiteboard to document key questions or areas for additional reflection and work. The results from the listening sessions mirrored those found in the survey data.

Considering the information gleaned from the staff surveys and listening sessions, the Next Steps workgroup drafted an outline of recommendations. Three key areas were identified for focused work: human resources, training, and church relations. The Core Team affirmed these three areas and nominated staff members to chair each workgroup. All staff in the agency were provided opportunities to sign up or to nominate another individual as a participant in the workgroups. Additionally, a communication plan was developed to ensure information from these groups was being shared.

The human resources team was charged with examining issues such as hiring and promotion practices, pay scales, and staff retention. Of note, the workgroup explored the demographic data of each department and found that primarily Black employees work in direct services while primarily white employees work in clinical roles. Additionally, the workgroup is reviewing employee satisfaction surveys and exit surveys to determine if any additional trends are emerging.

The training workgroup took responsibility for exploring how the agency is creating opportunities for staff to engage in both individual and social learning with regards to diversity, equity, and inclusion. To date, this workgroup has strived to ensure that all curriculum authored by the organization addresses cultural diversity. Additionally, a psychoeducational curriculum is in development that utilizes the SELF framework and explores various aspects of diversity and discrimination through the lens of the fundamental Sanctuary principles of safety, emotion management, loss, and future. Finally, each quarter all staff are invited to participate in a facilitated discussion that includes both an update on agency efforts and an opportunity for staff members to share their own experiences with the hope of creating more open dialogue and opportunities for authentic and empathic conversations.

The Church Relations Committee has focused on exploring the various ways faith and religion intersect for both clients and staff. According to a member of the group, “When we provide our young people the opportunity to explore their faith, we must be sure we are doing that in places they feel they can belong and grow. In order to create a spiritual safe-space for our kids, we need to be fully informed on what their church experience may consist of in relation to past trauma and current challenges.”

At the direction of the Core Team, the agency crafted a public statement about racism (see Appendix A). This statement was presented to and affirmed by the board of directors and can be found on the agency’s public website.

To encourage the Sanctuary principle of open communication and to ensure growth and change are taking place, the president/CEO of the organization began holding bi-monthly lunches with groups of staff members to develop systems to sustain organizational progress or to combat a lack of progression. This group has identified the following areas as potential for continued growth and change:

- More training needs to be implemented on cultural diversity to promote a better understanding of different cultures and different backgrounds, and to generate more creative solutions to specific problems.
- There needs to be an increase in the utilization of the seven commitments, especially open communication. Information needs to be transmitted comprehensively throughout the agency. People need to feel empowered to say what they mean, without being mean.
- Managers need to help foster morale in their teams by establishing trust, being accessible, and allowing open communication—listening to and learning from people.

The Sanctuary Model has provided The Methodist Home with a solid framework to aid in the creation of an anti-racist organizational culture. In particular, using key Sanctuary principles, such as the seven commitments and the SELF framework, has allowed the agency to approach such a significant undertaking through a trauma-informed lens while focusing on safety for all involved with the hope of continued growth and change. Although much work has been accomplished, the agency has acknowledged there will always be another step along the journey of creating a healthy, diverse, and equity-driven organization.

## 7. Discussion and Implications

Understanding the culture change that implementing the Sanctuary Model brings can help other human service organizations that are considering models of systems-based, trauma-informed culture change, specific to the work of cultural humility and anti-racism. The Sanctuary Model supports these endeavors through the adherence to the seven commitments, the organizational values to creating a trauma-informed community, allowing all voices to be heard and considered and providing opportunities to begin to repair previous experiences of inequity and suppression. Through the board of directors, leadership, and staff of a human services agency, an organization can transform the organizational culture into one that truly supports and embraces diversity, equity, and inclusion in its operations, in support of both staff and clients alike. 

This is evident in The Methodist Home’s use of the Sanctuary Model from the beginning of their Sanctuary journey to create a trauma-informed and responsive organization. The Methodist Home relied on the framework of the Sanctuary Model as it embarked on the work of understanding racism in its own agency, through a lens of cultural humility and a focus on eliminating racism. Leadership and the Core Team understood that they needed to hear from the staff to create a plan forward for the organization. Using the seven commitments to build on their environment of safety created over the past several years through Sanctuary Model implementation, along with the tools of the model, they were thoughtful in planning how they began and continued their journey. 

The Core Team was tasked with developing the plan, the next steps, for The Methodist Home to begin conversations and learn about the experiences of the Black employees of the organization. Equally important was the commitment to open communication on the part of the organization, whereby they analyzed and shared the data collected with all members of the community. These listening sessions for staff, conducted with outside moderators, supported the agency’s work toward cultural humility as a core competency to ensure that The Methodist Home is acknowledging diversity of knowledge, beliefs, and behaviors. The commitment to democracy is also evident in acknowledging the need to minimize power dynamics and keep the focus on the systemic topics of racism, discrimination, and White privilege, rather than delving into the particulars immediately. Knowledge of trauma theory, inclusive of re-enactment, also proved important as Black employees talked about situations that happened years and decades earlier, ones that still impacted their work today. In terms of internal structural changes, the next steps for The Methodist Home will focus on creating and amending policies to ensure alignment with their anti-racist efforts.

Given the success of The Methodist Home in using the Sanctuary Model as the framework for exploring cultural humility and racism at the organization, the Sanctuary Institute will continue to support organizations interested in this important work through further education of the faculty consultants and the development of training and workshops specifically focused on anti-racism and learning about the experiences of Black members of client organizations. In addition, there may be opportunities to conduct mixed-method studies to better understand the processes and outcomes of anti-racist organizational change efforts, as well as implications for oversight bodies, such as Social Current, the Joint Commission (JCAHO), and the Commission on Accreditation of Rehabilitation Facilities (CARF), to include cultural humility and anti-racist efforts as they review organizations for compliance with their standards of trauma-informed care. 

Anti-racism work is challenging and requires much commitment and focus of all members of an agency, especially those in leadership positions. Aligned with the Sanctuary commitments of growth and change, the field of trauma-informed services must include anti-racist organizational cultures so that staff who deliver services to clients, many from marginalized communities, are not marginalized themselves by the organizations they serve.

## Appendix A

### The Methodist Home’s Statement against Racism

In response to Christ and the church, the mission of The Methodist Home for Children and Youth is to be a model agency that restores childhoods, strengthens families, and cultivates a people-building organization.

After nearly 150 years of serving children and families in crisis, The Methodist Home is still committed to our mission by serving those who come to us in need of hope and healing. Today, we are deeply troubled and heartbroken by the recent events exposing racial injustices within our country and communities. We realize there have been too many years of inequities and systemic racism throughout the history of our nation and we recognize our Black sisters and brothers are hurting and want action now.

As a social services organization, we have learned a lot about trauma and how it can negatively impact individuals, families, and future generations. For this reason, we encourage compassion, social responsibility, emotional intelligence, nonviolence, and servant ministry. We strive to actively and honestly live in our faith and values every day and examine our own hearts.

Specifically, we are practicing open communication with our staff members and youth to address racism within our organization. We have initiated facilitated group conversations and online chapel services—inviting seasoned staff members to share their personal experiences with racism to help lead and guide us forward.

We believe Black lives not only matter, but Black lives are valuable and worthy of love and respect. It is our honor to stand with our Black youth, staff, and community members of color against racism and discrimination and we vow to continue looking inward as we grow to fully embrace anti-racism, equity, and justice for all.

## References

[1] Rigueur L.W., Beshlian A. (2019). The history and progress of black citizenship. Du Bois Rev. Soc. Sci. Res. Race.

[2] Patterson O. The Long Reach of Racism in the U.S. https://www.wsj.com/articles/the-long-reach-of-racism-in-the-u-s-11591372542.

[3] Solomon D., Maxwell C., Castro A. Systematic Inequality and American Democracy. https://www.americanprogress.org/issues/race/reports/2019/08/07/473003/systematic-inequality-american-democracy/.

[4] Brennan Center for Justice Voting Laws Roundup: May 2021. https://www.brennancenter.org/our-work/research-reports/voting-laws-roundup-may-2021.

[5] Cherry K. What Is Anti-Racism?. https://www.verywellmind.com/what-is-anti-racism-5071426.

[6] Koury S.P., Green S.A. Trauma-Informed Organizational Change Manual. http://socialwork.buffalo.edu/social-research/institutes-centers/institute-on-trauma-and-trauma-informed-care.html.

[7] Im H., Swan L.E.T. (2021). Working towards culturally responsive trauma-informed care in the refugee resettlement process: Qualitative inquiry with refugee-serving professionals in the United States. Behav. Sci..

[8] Sotero M.M. (2006). A conceptual model of historical trauma: Implications for public health practice and research. J. Health Disparities Res. Pract..

[9] Durham M., Webb S.S.N. (2014). Historical trauma: A panoramic perspective. Brown Univ. Child Adolesc. Behav. Lett..

[10] Braveheart M.Y.H. (2003). The historical trauma response among Natives and its relationship with substance abuse: A Lakota illustration. J. Psychoact. Drugs.

[11] Gump J.P. (2010). Reality matters: The shadow of trauma on African American subjectivity. Psychoanal. Psychol..

[12] Harrell S.P. (2000). A multidimensional conceptualization of racism-related stress: Implications for the well-being of people of color. Am. J. Orthopsychiatry.

[13] Nobles W.W. (2013). Shattered consciousness, fractured identity: Black psychology and the restoration of the African psyche. J. Black Psychol..

[14] Krysińska K., Lester D. (2006). The contribution of psychology to the study of the Holocaust. Dialogue Univers..

[15] Nagata D.K., Cheng W.J.Y. (2003). Intergenerational communication of race-related trauma by Japanese American former internees. Am. J. Orthopsychiatry.

[16] Administration for Children & Families What is Historical Trauma?. https://www.acf.hhs.gov/trauma-toolkit/trauma-concept.

[17] Chisolm R., Washington M. (1997). Undoing Racism: A philosophy of International Social Change.

[18] Pinderhughes E. (1989). Understanding Race, Ethnicity, and Power: The Key to Efficacy in Clinical Practice.

[19] Comas-Díaz L., Hall G.N., Neville H.A. (2019). Racial trauma: Theory, research, and healing: Introduction to the special issue. Am. Psychol..

[20] Carter R.T. (2007). Racism and psychological and emotional Injury: Recognizing and assessing race-based traumatic stress. Couns. Psychol..

[21] Center for the Study of Hate & Extremism (2018). Report to the Nation: Hate Crimes Rise in the U.S. Cities and Counties in Time of Division & Foreign Interference.

[22] Chou T., Asnaani A., Hofmann S.G. (2012). Perception of racial discrimination and psychopathology across three US ethnic minority groups. Cult. Divers. Ethn. Minority Psychol..

[23] The National Child Traumatic Stress Network. Culture and Trauma. https://www.nctsn.org/trauma-informed-care/culture-and-trauma.

[24] Black S.R., Spence S.A., Omari S.R. (2004). Contributions of African Americans to the field of psychology. J. Black Stud..

[25] Myers L.J., Speight S.L. (2010). Reframing mental health and psychological well-being among persons of African descent: Africana/Black psychology meeting the challenges of fractured social and cultural realities. J. Pan Afr. Stud..

[26] Peacock C., Daniels G. (2006). Applying an antiracist framework to a residential treatment center: Sanctuary^®^, a model for change. J. Emot. Abus..

[27] Danzer G., Rieger S.M., Schubmehl S., Cort D. (2016). White psychologists and African Americans’ historical trauma: Implications for practice. J. Aggress. Maltreatment Trauma.

[28] Alleyne A. (2004). Black identity and workplace oppression. Couns. Psychother. Res..

[29] Alleyne A. (2004). The internal oppressor and black identity wounding. CPJ Couns. Psychother. J..

[30] Wilson W. (2009). More than just Race: Being Black and Poor in the Inner City.

[31] Hormann S., Vivian P. (2005). Toward an understanding of traumaized organizations and how to intervene in them. Traumatology.

[32] Couto R.A. (1989). Redemptive organizations and the politics of hope. Clin. Sociol. Rev..

[33] Hirschhorn L. (1988). The Workplace within: Psychodynamics of Organizational Life.

[34] Schulman M., Gingrich M. Cultural Humility: A Key Element of Trauma-Informed Care. https://www.chcs.org.

[35] Meléndez Guevara A.M., Lindstrom Johnson S., Elam K., Hilley C., McIntire C., Morris K. (2021). Culturally responsive trauma-informed services: A multilevel perspective from practitioners serving latinx children and families. Community Ment. Health J..

[36] Akerele O., McCall M., Aragam G. (2021). Healing ethno-racial trauma in black communities: Cultural humility as a driver of innovation. JAMA Psychiatry.

[37] Hanson R.F., Lang J. (2016). A critical look at trauma-informed care among agencies and systems serving maltreated youth and their families. Child Maltreatment.

[38] Gottlieb M. (2020). The case for a cultural humility framework in social work practice. J. Ethn. Cult. Divers. Soc. Work.

[39] Blitz L.V., Illidge L.C. (2006). Not so black and white: Shades of gray and brown in antiracist multicultural team building in a domestic violence shelter. J. Emot. Abus..

[40] Bloom S. (2007). The Sanctuary Model of trauma-informed organizational change. Source Natl. Abandon. Infants Assist. Resour. Center..

[41] Bloom S.L., Sreedhar S.Y. (2008). The Sanctuary Model of trauma-informed organizational change. Part Spec. Issue Trauma Trust.

[42] Esaki N., Benamati J., Yanosy S., Middleton J.S., Hopson L.M., Hummer V.L., Bloom S.L. (2013). The Sanctuary Model: Theoretical framework. Fam. Soc..

[43] Bills L.J. (2003). Using trauma theory and S.A.G.E. In outpatient psychiatric practice. Psychiatr. Q..

[44] Bloom S.L., Farragher B. (2011). Destroying Sanctuary: The Crisis in Human Service Delivery Systems.

[45] Bloom S.L. (2013). Creating Sanctuary: Toward the Evolution of Sane Societies.

[46] Bloom S.L. (1997). Creating Sanctuary.

[47] Abramovitz R., Bloom S.L. (2003). Creating Sanctuary in residential treatment for youth: From the "Well-ordered Asylum" to a "Living-Learning Environment". Psychiatr. Q..

[48] Sanctuary Institute Certification. https://www.thesanctuaryinstitute.org/services/certification/.

